# Deep Learning Techniques and Imaging in Otorhinolaryngology—A State-of-the-Art Review

**DOI:** 10.3390/jcm12226973

**Published:** 2023-11-08

**Authors:** Christos Tsilivigkos, Michail Athanasopoulos, Riccardo di Micco, Aris Giotakis, Nicholas S. Mastronikolis, Francesk Mulita, Georgios-Ioannis Verras, Ioannis Maroulis, Evangelos Giotakis

**Affiliations:** 1 1st Department of Otolaryngology, National and Kapodistrian University of Athens, Hippocrateion Hospital, 115 27 Athens, Greece; arisgiotakis@gmail.com (A.G.); giotakis@gmail.com (E.G.); 2 Department of Otolaryngology, University Hospital of Patras, 265 04 Patras, Greece; miathanasopoulos@gmail.com (M.A.); nmastr@otenet.gr (N.S.M.); 3 Department of Otolaryngology and Head and Neck Surgery, Medical School of Hannover, 30625 Hannover, Germany; rdm1331991@gmail.com; 4 Department of Surgery, University Hospital of Patras, 265 04 Patras, Greece; georgiosverras@gmail.com (G.-I.V.); maroulisupatras@gmail.com (I.M.)

**Keywords:** otorhinolaryngology, deep learning, artificial intelligence, convolutional neural network, computer vision, imaging

## Abstract

Over the last decades, the field of medicine has witnessed significant progress in artificial intelligence (AI), the Internet of Medical Things (IoMT), and deep learning (DL) systems. Otorhinolaryngology, and imaging in its various subspecialties, has not remained untouched by this transformative trend. As the medical landscape evolves, the integration of these technologies becomes imperative in augmenting patient care, fostering innovation, and actively participating in the ever-evolving synergy between computer vision techniques in otorhinolaryngology and AI. To that end, we conducted a thorough search on MEDLINE for papers published until June 2023, utilizing the keywords ‘otorhinolaryngology’, ‘imaging’, ‘computer vision’, ‘artificial intelligence’, and ‘deep learning’, and at the same time conducted manual searching in the references section of the articles included in our manuscript. Our search culminated in the retrieval of 121 related articles, which were subsequently subdivided into the following categories: imaging in head and neck, otology, and rhinology. Our objective is to provide a comprehensive introduction to this burgeoning field, tailored for both experienced specialists and aspiring residents in the domain of deep learning algorithms in imaging techniques in otorhinolaryngology.

## 1. Introduction

Artificial intelligence (AI) refers to the simulation of human intelligence in computer systems. It involves the development of algorithms and models that enable machines to perform tasks that typically require human intelligence, such as problem-solving, learning from experience, recognizing patterns, and making decisions. AI encompasses various subfields, including machine learning, natural language processing, computer vision, and robotics, all aimed at creating systems that can mimic human cognitive functions and behaviors [1].

These new technologies have been evolving during the last decades in all areas of medicine, including otorhinolaryngology, but it was the COVID-19 pandemic that led to the widespread adoption of such novel tools. During that time, AI applications assisted in increasing consciousness regarding the health and safety of both patients and healthcare practitioners and in driving behavioral alterations. At the same time, more sophisticated and accurate algorithms were developed [2].

The next contribution that complex AI systems promise is the IoMT. The recent COVID-19 pandemic but also the demands of everyday life can provoke inconvenience in visiting healthcare facilities for minor health issues. An IoMT system, comprising different interconnected medical devices through the internet, facilitates medical monitoring [3]. Basic IoMT architectures require the acquisition of patient medical information through smart sensors embedded in wearable devices. These devices are interconnected through a body sensors network (BSN) or a wireless sensor network (WSN), and the collected data are transmitted via the internet to the next stage, where analysis and data evaluation take place through delicate AI algorithms. The final step is medical intervention in case of a possible serious medical issue [4].

**Figure 1:**
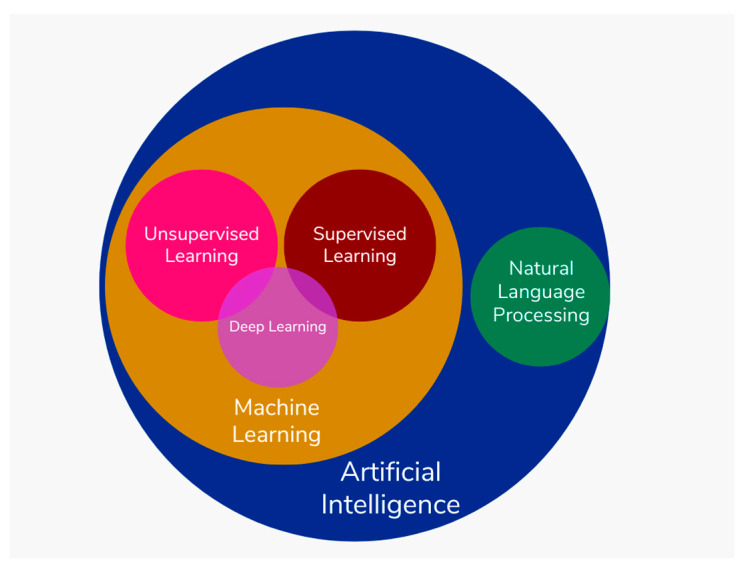
Categorization of AI systems. Deep learning systems consist of a subcategory of machine learning algorithms.

Machine learning techniques consist a large category of AI models. These systems are granted the ability to learn and enhance themselves through experience, gradually becoming adept at performing specific tasks, and human involvement remains necessary during their training phase. A categorization of AI systems is depicted in Figure 1. 

Novel deep learning (DL) systems, a subset of machine learning models, employ intricate algorithms and neural networks with multiple complex layers for training, but often require a significant direct human input [5,6]. The tasks that these algorithms undertake include, in general, intricate computations [7], predictions [8], and repetitive analytical activities [9]. The combination of computer vision with DL applications presents the capability to manage extensive medical image datasets, enabling precise and effective diagnosis. Additionally, it has the potential to mitigate the considerable intra- and inter-observer variability, which can compromise the reliability of clinical assessments [10].

Deep learning systems, as revolutionary tools in the field of medical imaging, have significantly enhanced diagnostic accuracy and efficiency. These systems employ intricate neural networks to autonomously analyze complex medical images in the head and neck area, such as CT scans, MRIs, PET-scans, and U/S images. 

This wide area of available techniques offers several possibilities, spanning from improving the quality of medical images and segmentizing specific structures or lesions to detecting anomalies. Their ability to detect patterns and abnormalities within images has led to the early and precise identification of various conditions, including both benign and malignant diseases. Moreover, deep learning algorithms continually refine their performance through exposure to vast amounts of medical data, making them increasingly adept at recognizing subtle variations that might escape human observation. As a result, these systems hold the potential to revolutionize diagnostics and decision-making in otorhinolaryngology, offering ear, nose, and throat (ENT) specialists invaluable support in delivering timely and accurate diagnoses to improve patient outcomes.

The future significance of AI in the practice of medicine, and specifically in our specialty, is undisputed, and thus it is deemed, nowadays, necessary for the otolaryngologists to be familiar with the concepts and the existing techniques in computer vision and DL algorithms. The goal of this narrative review is to serve as an introduction, for the specialists and residents in ENT medicine, to the domain of these models. To our knowledge, this is the first paper addressing this issue in its entirety, in terms of a review.

## 2. Head and Neck

### 2.1. Head and Neck Imaging

Head and neck surgery relies majorly on imaging, which is often a pre-requisite before any further management. Different techniques offer significant advantages in disease diagnosis but also follow-up. Computed tomography (CT) and magnetic resonance imaging (MRI), during the last decades, have usually been used combinatorically in a large variety of medical conditions to acquire both bone and soft tissue information. 

Lately, deep learning algorithms have emerged, which enable the conversion of one imaging modality to another. For example, MRI scans, which involve bone techniques, give us the possibility of a subsequent MRI to CT reconstruction, avoiding exposure to ionizing energy and aiding non-experts in diagnosis at the same time [11]. A combination of two generative adversarial networks has also been implemented to generate accurate synthetic CT images from MRI scans [12]. On the other hand, non-contrast CT scans can be converted to PET-like images with generative models, eliminating the need for radioactive tracers. The generated PET images demonstrate comparable accuracy to actual FDG-PET images in predicting clinical outcomes [13]. It seems rational to hypothesize that such deep learning pipelines can transform head and neck imaging into a one-step-procedure in the future.

Next, CNNs are believed to exhibit superior performance compared to a traditional radiomic framework regarding their ability to detect image patterns, often undetectable by the latter, while systems such as ultra-high-resolution CT with a DL-based image reconstruction engine offer significant amelioration in subjective and objective image quality, with a higher sound-to-noise ratio, lower noise, and lower radiation exposure [14].

The DL technique utilized in the analysis of medical images allows the incorporation of both qualitative and quantitative imaging characteristics to create prediction models characterized by exceptional diagnostic accuracy. These principles have been applied generally to HNSCC imaging, but also specifically to specific types of HNSCC. Notably, in the imaging of oral and oropharyngeal cancer, FDG-PET/CT scans can be processed by DL systems to predict local treatment outcomes [15], disease-free survival with high sensitivity and specificity [16], overall survival [17], and they can even assist in differentiating human papillomavirus positive from human papillomavirus negative oropharyngeal carcinomas [18].

At the same time, progress in computer vision and deep learning provide potent techniques for creating supplementary tools capable of automatically screening the oral cavity. These cost-effective and non-invasive tools can offer real-time insights for healthcare practitioners during patient assessments and can also facilitate self-examinations for individuals. The automated diagnosis of oral cancer through images is predominantly focused on the utilization of specialized imaging technologies, namely optical coherence tomography [19,20], hyperspectral imaging [21], and autofluorescence imaging [22], but also white-light photographs [23]. Such DL techniques can come in the form of mHealth applications, assisting in oral and oropharyngeal lesion detection in both hospitals and resource-limited areas, and enabling telediagnosis [24]. Finally, systems offering a real-time estimation of cancer risk and biopsy assistance maps on the oral mucosa are very promising [25]. 

Furthermore, diseases of the nasopharynx have been an area of focus during the last years for DL system developers. From MRI-based applications focusing on the differential diagnosis between benign and malignant nasopharyngeal diseases [26,27] to the automatic detection of pathological lymph nodes and assessment of the peritumoral area in nasopharyngeal carcinoma, DL algorithms can significantly assist in disease prognosis and treatment planning [28]. Interestingly, peritumoral information, especially the largest areas of tumor invasion, has been shown to provide valuable insights for distant metastasis prediction in individuals with nasopharyngeal carcinoma [29].

Imaging of the salivary glands constitutes another significant challenge for radiologists and otolaryngologists, who have many different imaging modalities in their quiver. Specialized DL algorithms have been developed to assist in differential diagnosis between benign and malignant parotid gland tumors in contrast-enhanced CT images [30], and ultrasonography [31]. ΜRI remains the gold standard in the diagnosis of salivary gland diseases, where DL models intend to automatically classify salivary gland tumors with very high accuracy [32,33].

Relative to thyroid disease diagnosis, ultrasound (US) is widely acknowledged as the primary diagnostic technique for examining thyroid nodules and assessing papillary thyroid carcinomas (PTCs) before surgery [34]. DL networks with excellent diagnostic efficiency have been deployed to distinguish between benign nodules and thyroid carcinoma [35], improve the detection of follicular carcinoma, differentiate between atypical and typical medullary carcinoma [36], and assess for gross extrathyroidal extension in thyroid cancer [37]. AI systems can be very useful in eliminating the operator dependence of US and ameliorating diagnosis precision, especially in inexperienced radiologists. 

Nevertheless, plenty of other DL techniques are associated with thyroid gland evaluation. Thus, apart from thyroid gland contouring in non-contrast-enhanced CT images [38], special applications used intraoperatively to assist surgeons in recurrent laryngeal nerve [39] and parathyroid gland identification have been designed. Such algorithms have the potential to improve surgical workflows in the intricate environment of open surgery. 

The head and neck region is among the most common locations for cancer, with a substantial occurrence of lymph node involvement and metastases observed in both nearby and distant regions. The identification of distant metastases is linked to an unfavorable prognosis, often resulting in a median survival period of around 10 months [40]. The role of imaging in metastasis diagnosis is uncontroversial and novel convolutional neural networks have been developed in this direction. For example, extended 2D-CNN and 3D-CNN models have been deployed to perform time-to-event analysis for the binary classification of distant metastasis in head and neck cancer patients. These models result in the generation of distant metastasis-free probability curves and stratify patients into high- and low-risk groups [41]. CNN are generally able to detect image patterns that can be untraceable with traditional methods. Thus, it has been shown that CNN can be trained to forecast the treatment results for individuals with HNSCC, relying exclusively on the information from CT scans conducted prior to treatment [42]. 

CNN assessing pre-treatment MRI scans to predict the possibility of distant metastases in individuals with nasopharyngeal carcinoma can also be useful, since the occurrence of a metastasis is the main reason for radiotherapy failure in this patient group. Predicting the high risk for distant metastasis in a patient can lead to a more aggressive treatment approach [29]. Moreover, pre-therapy MRI scans have been used in patients with advanced (T3N1M0) nasopharyngeal carcinoma to guide the clinicians in deciding between induction chemotherapy plus concurrent chemoradiotherapy or concurrent chemoradiotherapy alone [43].

DL models diagnosing lymph node involvement can boost clinical decision-making in the future. A relative model has been developed that detects pathological lymph nodes in individuals with oral cancer [44], while another one predicts lymph node involvement in patients with thyroid cancer through the interpretation of their multiphase dual-energy spectral CT images [45].

The utilization of deep learning techniques allows for the complete automation of image analysis providing the user with multiple possibilities (Table 1). Nevertheless, it demands a substantial volume of accurately labeled images. Additionally, prediction-making necessitates detailed patient endpoint data, a process that is both expensive and time-intensive. Developing more effective models with constrained datasets stands as a critical challenge in the field of AI today. 

### 2.2. Head and Neck Radiotherapy

Radiotherapy (RT) stands as a fundamental pillar in head and neck cancer (HNC) treatment, whether administered independently, post-surgery, or concurrently with chemotherapy. Defining organs at risk (OARs) and clinical target volumes represents a crucial phase in the treatment protocol. This process typically involves manual work, is time-consuming, and necessitates substantial training. Ideally, these tasks would be substituted by automated procedures requiring minimal clinician involvement, and AI appears competent to undertake this role. 

A major challenge and the primary drawback of radiation therapy is that, apart from the cancerous mass, it unavoidably exposes nearby healthy tissues, known as OARs, to some level of radiation. This can potentially result in various adverse effects and toxicities, since contouring organs like the parotid and the submandibular gland and excluding them from radiation intake can be quite arduous [46]. Additionally, DL-based automated segmentation of the masticatory area has successfully reduced the incidence of RT-associated trismus [47].

Several applications aiming to realize normal tissue structure auto-segmentation from CT images [48,49] exist. These can include three-dimensional segmentation models and convolutional neural networks for final OAR identification [50]. DL pipelines focusing on tumor segmentation in specific organs, such as the oropharynx [51], and the salivary glands promise to gradually automatize the RT procedure, and at the same time reduce post-segmentation editing [52].

On the other hand, 3D CNNs aim to consistently and precisely generate clinical target volumes contouring for the different lymph node levels in HNSCC RT [53,54]. Such applications show quicker contouring adjustments in comparison to automated delineations, closely aligning with corrected delineations for specific levels, and reducing interobserver variability.

The possibilities that DL systems offer are countless, with distant metastasis and overall survival prediction in HNSCC using PET-only models without gross tumor volume segmentation [55], or automatically delineating the gross tumor volume in the FDG-PET/CT images of HNSCC patients [56]. Overall, DL systems present the potential to offer personalized RT guidance in HNSCC patients, with limited contribution from medical experts. 

### 2.3. Endoscopy and Laryngoscopy

Machine learning has been recently experimentally applied to diagnostic ENT endoscopy to leverage meaningful information from digital images, videos, and other visual inputs and take actions or make recommendations based on that information. Mediated from the early experience acquired in the more standardized field of gastrointestinal endoscopy, AI-based video analysis, or videomics [57], has been variously applied to improve automatic image quality control, classification, optical detection, and the segmentation of images. After numerous proof-of-concept studies, videomics is rapidly moving to viable clinical approaches for detecting pathological patterns in real-time assistance during the endoscopic evaluation of the upper aerodigestive ways.

A deep learning model consists of complex multilayer artificial neural networks, among which convoluted neural networks are the most popular in the image analysis field. The CNN does not require instructions on which features describe an object and can autonomously learn how to identify it by observing a sufficient number of examples. Various available AI models exist and have been applied [58], although a specific comparison between the various algorithm architectures for the task is still lacking. After this preliminary conceptualization phase, the model undergoes a supervised learning session, in which expert otolaryngologists provide the AI human annotated images to transfer their ability in recognizing the lesions. The higher the quality and quantity of items in the validation set, the more accurate the model will be. After the training validation set, the performance of the system is measured on the testing set by comparing the model prediction with the original human annotations. The performance will be evaluated using diagnostic metrics relative to the task analyzed. 

AI can be used to classify endoscopic images. In that case, the diagnostic metrics of interest are accuracy (percentage of correctly classified images), precision (positive predictive value), and sensitivity (percentage of correctly identified images compared to all the ones that should have been recognized); F1 score (harmonic mean of precision and sensitivity); and the receiver operating characteristic curve (graphically identifying the true positive rate against the false positive one) [59]. In this framework, it is possible to apply AI to classify videos based on their image quality, selecting only the most informative frames for further analysis [60,61]. Another classification task is the optical biopsy [62], predicting the histology of a lesion based on its appearance. At the current state, AI is more accurate in binary classification, e.g., premalignant/malignant [63], whereas it loses diagnostic power in multiclass operation [64]. By expanding and diversifying the validation dataset, it is possible to achieve high accuracy in simultaneously identifying different conditions such as glottic carcinoma, leucoplakia, nodules, and vocal cord polyps [65], outperforming other approaches according to AUC and F1 otolaryngologist trainees [66]. 

Another task the AI is devised for is the automatic detection of lesions during endoscopic evaluation. The main diagnostic metrics for this function are the F1 score, the intersection over union (how well the selected area overlaps with the original annotated area), and the mean average precision (precision and sensitivity according to the chosen IoU). Using narrow band images, AI can be trained to localize mucosal cancerous lesions in the pharynx and larynx during endoscopy [67,68,69]. This concept has been recently applied to automatically detect laryngeal cancer in real time video-laryngoscopy using the open-source YOLO CNN, achieving 67% precision, 62% sensitivity, and 0.63 mean average precision at 0.5 IoU [70], which could be implemented in a self-screening approach for early tumor recurrence detection [71]. Based on simple diagnostic endoscopy, the same approach can be applied intraoperatively to detect pathological tissues, such as in endoscopic parathyroid surgery [72,73].

Finally, CNN has been used to automatically delineate the boundaries of anatomical structure and lesions in the upper aerodigestive ways. Segmentation performance is evaluated with IoU and the dice similarity coefficient (similarity between the predicted segmentation mask and the ground truth mask). The rationale of segmentation in videomics is to improve lesion follow-up, the definition of tumor resection margins in the operation room, and the area of interest for general laryngology. The automated segmentation of cancer tissue has been successfully attempted in the nasopharynx (DSC 0.78) [74], oropharynx (DSC 0.76) [75], and laryngeal lesion (DSC 0.814) [76]. Aside from cancer pathology, segmentation may be used to select the region of interest for automated functional laryngeal analysis, such us the identification of the glottis angle [77,78], glottal midline [79], vocal cord paralysis [80], postintubation granuloma [81], vocal cord dynamics [82,83], or in the endoscopic evaluation of aspiration and penetration risk in dysphagia (FESS-CAD, DSC 0.92.5) [84].

Building a sufficiently large and heterogeneous training image dataset is a necessary task required to improve the deep learning-based image classifier. The main obstacles remain the lack of standardization of endoscopic techniques and study structures, hampering a comparison between the different experiences, and the complex anatomy of the upper aerodigestive ways, making image acquisition and standardization difficult. Although deep learning models can be very good at analyzing images belonging to the same group of the training cohort, they may lack accuracy when tested on different populations. To effectively apply videomics in real world situations, future research should focus on validating the trained models with an external dataset, acquired in different institutions and thus being diverse in terms of acquisition technique and population demographics. Although AI-aided endoscopy is still in a preclinical state, the results are promising and may soon efficiently assist the otolaryngologist in many tasks, such as the quality assessment of endoscopic examination, detection of mucosal lesions during endoscopy, optical biopsy of selected lesions, segmentation of cancer margins, and the assessment of laryngeal mobility.

## 3. Otology 

### 3.1. Computer Vision in Otoscopy

The otoscopic ear inspection remains the first and most important step in the diagnosis of ear disease, especially otitis media and its variants. However, otoscopy requires extensive training, and there are still high rates of errors even with experienced otolaryngologists [85]. The growing use of video-otoscopy provides reliable data for developing deep learning models for automated image recognition, potentially assisting the less experienced physician in the identification and classification of pathological findings [86]. 

The most common machine learning approach used in automatized video-otoscopy is the convolutional neural network, a type of deep learning model which undergoes a supervised learning session using human-labeled data to master in order to recognize specific pathological patterns in a dataset of retrospectively collected images. CNN can be used for image classification, detection, and segmentation. As in videomics, one application is the optimization of the diagnostic image, such as the selection of the best quality image frames in an otoscopic video recording in order to create an informative composite image, stitching together only the best quality frames and excluding the less informative ones [87]. Moreover, CNN can provide an automatized segmentation of the eardrum from otoscopic images, orienting the clinician and future CNN to special areas of interest for the diagnosis [88]. Automatic image preprocessing, reducing imperfections such as motion artifacts or earwax [89], and selecting the proper color wavelength [90] may further enhance the informativeness of the pictures. Once the images have been properly selected, the final step of building an AI image classifier requires training on a large, annotated image dataset and validation of the results. The accuracy of the system is usually determined by a comparison with the diagnosis of a panel of experienced physicians or otolaryngologists. AI image classifiers have been mostly studied for the automatic diagnosis of otitis media. Many different available CNN models have been variously trained and compared (ResNet-50, Inception-V3, Inception-Resnet-V2, MobileNetV2) to binarily differentiate between normal and abnormal images [91] or to attempt multiclass classification [92,93,94,95], achieving on average a 90.47% accuracy in differentiating between normal and abnormal images and 97.6% between normal, otitis media acuta, and otitis media with effusion [96]. AI algorithms experimentally outperformed human assessors in classifying otoscopy images, achieving 93.4% versus 73.2% accuracy [93,94,97,98]. The same approach has been investigated for other otologic conditions, such as eardrum perforation [99], attic atelectasis [100], and otomycosis [101]. Comparing different studies and approaches is, however, difficult for the heterogeneity of the collected dataset, making the standardization of otoscopic image acquisition and annotation an important step for future developments [102]. 

It is also possible to apply deep learning models, which cluster data together based on similarity to provide predictions and reveal common themes, to make clinical predictions based on the collected images. For example, the optical recognition of pathological tympanic membranes can be paired with hearing loss predictions. In a preliminary study, a deep learning algorithm created to analyze video pneumatic otoscopy images accurately detected the presence of conductive hearing losses caused by middle ear effusion, ossicular fixation, otosclerosis, and adhesive otitis media, outperforming experienced otologists [103]. Similarly, CNN proved better than clinicians and logistic regression models in predicting a conductive hearing loss greater than 10 dB, focusing on the retraction pockets in otitis media images [104]. 

Although experimentally AI image classifiers can achieve accuracies comparable to those of experienced otologists, separately trained CNN still fails to maintain the same high internal performance when applied to a different cohort from the one used for training, although organized in the same way, as demonstrated in a multicenter study [105]. The accuracy of CNN image classifiers heavily relies on the quality and quantity of the image dataset used for training. Not having access to big quality data remains a persistent hindrance in many pilot studies; consequently, internal solutions such as data augmentation with rotation and cropping have been generally applied, with still-debatable consequences. A possible solution to the problem is represented by transfer learning procedures, in which knowledge learned from a task is extracted and re-used to boost performance in a related task. A CNN pretrained on another huge image database performs better in a small number of subjects as compared to one starting from scratch [106]. Potentially, creating a shared virtual imaging database could offer a better training dataset for future deep learning models, enhancing accuracy even in real-world applications. Although the bulk of the available literature is still at an infancy level in terms of practical implementation, the experimental outcomes have overall diagnostic accuracies not inferior to those of an experienced clinician.

### 3.2. Imaging in Otology

Deep learning is poised to integrate and assist the clinician in complex diagnosis by identifying patterns often imperceptible to humans, providing innovative health care solutions, especially in the field of telemedicine and early diagnosis. Artificial intelligence applications in otology are rapidly moving from simple proofs of concept to preliminary clinical applications in the field of applied radiomics.

A promising application of artificial intelligence in otology is the automatic segmentation and analysis of specific radiological images of the temporal bone. As in other cases of radiomics, the current approach consists of training the AI to automatically identify regions of interest based on sets of training images previously annotated by experienced radiologists. In this case, the measure of interest is the dice score, which compares the manual segmentation from human experts with the AI ones. 

Highly accurate automatic segmentation of radiological images has been demonstrated in CT scans [107], MRI scans [108] and cone-beam scans [109,110]. After selecting the region of interest, deep learning models can be specifically trained to classify specific diseases, such as chronic otitis media [111], cholesteatoma [112,113], otosclerosis [114,115], mastoiditis [116,117], and Meniere disease [118,119], achieving detection results comparable to subspeciality-trained radiologists. AI-assisted radiomics can be extremely useful in the follow-up of specific diseases, such as vestibular schwannoma, whose surveillance is nowadays performed through analogical segmentation and an analysis of serial MRI scans to detect tumor enlargement. A deep learning approach can be applied for tumor detection and segmentation in treatment-naïve patients [120], both after radiosurgery [121], in evaluating residual disease [122], and in predicting tumor enlargement based on radiomics parameters during follow-up [123]. 

Although highly successful in experimental settings, all these studies are currently performed only on a small number of patients for AI standards, making real-world clinical applications challenging. There is a growing need for image collection standardization and a multicenter approach to pull more different data together to better approximate real-life situations.

Although promising and quickly expanding (Table 2), the use of AI in otology is still associated with difficult translation in clinical practice. As soon as the trained AI is applied to a different group of patients or data as compared with the experimental setting, the prediction accuracy decreases. The greatest limitation remains that AI relies on training using a massive dataset. To build a sufficiently large and reliable database to encompass a real-world clinical situation is challenging and time consuming in a still unstandardized clinical practice. Another obstacle is the difficulty encountered in interpreting how the AI draws its conclusions, as it is impossible to evaluate which features are used by the AI to make its predictions. The learning mechanisms remain mostly unclear. 

## 4. Imaging in Rhinology

The field of rhinology, as a subspecialty, has witnessed numerous technological advancements, from endoscopic diagnosis and the treatment of paranasal diseases some decades ago to innovations like image-guided surgical navigation more recently. In an effort to provide personalized treatment and ameliorate surgical practice and accuracy, it is no wonder that a growing amount of research has focused on computer vision in rhinologic diseases (Table 3). 

Imaging in rhinology concentrates a plethora of DL systems aiming to augment diagnostic accuracy in particular domains of plain radiography, CT, and MRI imaging. As a general rule, the dependability of radiography in assessing sinusitis is debatable, since the documented sensitivity is relatively low for all sinuses, except for maxillary sinusitis. An algorithm capable of identifying and categorizing individual paranasal sinuses using both Waters’ and Caldwell’s views, all without requiring manual cropping, is available and could be useful, especially in areas and health facilities with limited resources [124]. In another study, panoramic imaging was applied to help dentists diagnose maxillary sinusitis [125]. Additionally, a generative adversarial network system offers amelioration of the diagnostic efficacy of sinus radiography since it requires considerably less real healthcare datasets [126]. 

Computed tomography imaging, which constitutes the gold standard in the imaging of the paranasal sinuses, presents a variety of challenges that DL algorithms are called upon to face. Firstly, preoperative sinus CT scans have been utilized to train a system to differentiate between non-eosinophilic and eosinophilic chronic rhinosinusitis (CRS), relying solely on CT imaging [127]. Second, CRS presents a tendency to recur and poor prognosis even following surgery. DL techniques aim to confront this problem by predicting, pre-operatively, the risk of disease recurrence [128]. Such a solution could augment patient-oriented therapy modalities.

Chronic rhinosinusitis (CRS) constitutes a diverse range of conditions defined by chronic inflammation of the paranasal sinuses. Although clinical examination has a key role in the diagnosis of the disease, CT is of vital importance in appraising sinusitis. Thus, the need for objective, enhanced, and standardized evaluation has led to the development of systems that realize the prompt evaluation of the paranasal sinuses’ opacification on CT images in patients with chronic rhinosinusitis [129,130]. The efficacy of these algorithms is strongly correlated to the Lund–Mackay score since they are, as the Lund–Mackay score, moderately correlated to the Lund–Kennedy endoscopy score [130]. Similarly, a CNN system can assess occlusion of the osteomeatal complex in individuals with chronic rhinosinusitis, relying on coronal CT images [131]. 

Applications evolving MRI techniques have also been developed, with the example of a three-dimensional CNN which can differentiate between benign and malignant inverted papilloma [132].

## 5. Conclusions

DL systems utilize complex algorithms and neural networks featuring numerous intricate layers in order to make decisions and solve advanced problems. Their application in medicine, and specifically in otorhinolaryngology, has increased rapidly, with a plethora of different and usually overlapping algorithms appearing in each and every subspecialty of ENT surgery. Due to their already wide utilization in everyday clinical practice, which is expected to rise expeditiously during the coming years, the modern otolaryngologist is obliged to be aware of and familiar with their multiple utilities.

## Figures and Tables

**Table 1:** The contributions of deep learning systems in head and neck imaging and radiotherapy.

Deep Learning Contributions in Head and Neck	
Imaging	Generation of an imaging modality from another
	Prediction making based on imaging
	Automated diagnosis of malignant and benign diseases
	Automated diagnosis of pathological lymph nodes
	Automated diagnosis of metastases
	Analysis of specific tumor characteristics
	Contouring of significant structures
	Cancer risk assessment of a lesion
	Biopsy assistance mapping
	Intraoperative surgeon assistance
Radiotherapy	Auto-segmentation of structures based on imaging
	Automation of the procedure
	Automated clinical target volume contouring
Endoscopy and laryngoscopy	Image quality improvement
	Segmentation of images
	Optical detection
	Pathological pattern detection
	Endoscopic image classification
	Lesion histology (benign/malignant) prediction
	Self-screening tumor recurrence detection
	Intra-operative endoscopic lesion detection
	Anatomical structure and lesion automatic segmentation
	Automatic assessment of aspiration and dysphagia
	Evaluation of laryngeal mobility

**Table 2:** The contributions of deep learning techniques in otology.

Deep Learning Contributions in Otology	
Otoscopy	Automatic detection and categorization of ear lesions
	Automatic segmentation of anatomical structures and lesions
	Optimization of the diagnostic images
	Lesion-based predictions
Imaging	Complex pattern identification
	Tele-diagnosis
	Automatic analysis and segmentation of images
	Automatic recognition of region of interest
	Automatic diagnosis
	Imaging follow-up of complex diseases

**Table 3:** The contributions of deep learning systems in rhinology.

Deep Learning Contributions in Rhinology
Identification and categorization of paranasal sinuses
Diagnosis of benign/malignant lesions
Prediction of disease recurrence
Detection of pathology related to chronic sinusitis
